# Deep proteome investigation of high-grade gliomas reveals heterogeneity driving differential metabolism of 5-aminolevulinic acid

**DOI:** 10.1093/noajnl/vdad065

**Published:** 2023-06-16

**Authors:** Saicharan Ghantasala, Amruth Bhat, Unnati Agarwal, Deeptarup Biswas, Prawesh Bhattarai, Sridhar Epari, Aliasgar Moiyadi, Sanjeeva Srivastava

**Affiliations:** Centre for Research in Nano Technology and Sciences, Indian Institute of Technology Bombay, Mumbai, India; Centre for BioSystems Science and Engineering, Indian Institute of Science, Bengaluru, India; Department of Biotechnology, School of Bioengineering and Biosciences, Lovely Professional University, Phagwara, India; Department of Biosciences and Bioengineering, Indian Institute of Technology Bombay, Mumbai, India; School of Biosciences and Technology, Vellore Institute of Technology, Vellore, India; Department of Pathology, Tata Memorial Centre’s—Advanced Centre for Treatment, Research and Education in Cancer, Navi Mumbai, India; Homi Bhabha National Institute, Mumbai, India; Homi Bhabha National Institute, Mumbai, India; Department of Neurosurgery, Tata Memorial Centre’s—Advanced Centre for Treatment, Research and Education in Cancer, Navi Mumbai, India; Department of Biosciences and Bioengineering, Indian Institute of Technology Bombay, Mumbai, India

**Keywords:** 5-ALA, deep proteome, GSEA, heme biosynthesis, HR LC–MS

## Abstract

**Background:**

Fluorescence-guided surgery (FGS) using 5-aminolevulinic acid (5-ALA) as adjunct for high-grade gliomas (HGGs) has been on the rise in recent years. Despite being largely effective, we observed multiple histologically similar sub-regions of the same tumor from a few individuals with varying protoporphyrin IX (PpIX) levels. The current study aims at understanding the proteomic changes driving differential metabolism of 5-ALA in HGGs.

**Methods:**

Biopsies were histologically and biochemically assayed. Following this, a deep proteomics investigation was carried out using high resolution liquid chromatography—mass spectrometry (HR LC-MS) to identify protein expression in differentially fluorescing regions of HGGs.

**Results:**

Our analysis identified 5437 proteins with high confidence. Differential analysis in the subgroup with HGGs carrying IDH mutation (IDH mt.) revealed 93 differentially regulated proteins (raw p-value ≤ 0.05 and absolute FC ≥ 1.5). Similar analysis in the IDH wild type (IDH wt.) subgroup revealed 20 differentially regulated proteins. Gene set enrichment analysis (GSEA) identified key pathways like ion channel transport, trafficking of AMPA receptors, and regulation of heme-oxygenase-1 in the IDH wt. subgroup. Pathways such as scavenging of heme, signaling by NOTCH4, negative regulation of PI3-AKT pathway, and iron uptake and transport were observed to be differentially regulated in the IDH mt. subgroup.

**Conclusions:**

Tumor regions from the same patient exhibiting differential fluorescence following 5-ALA administration were observed to have different proteome profiles. Future studies aimed at a better molecular understanding of 5-ALA metabolism in HGGs hold the potential to increase the efficacy of FGS and the use of 5-ALA as a theragnostic tool.

**Key Points**

- First LC–MS/MS-based proteomics study investigating differences in proteome profiles in tumors resected using 5-ALA fluorescence-guided surgery.
- Differentially fluorescing regions from the same tumor exhibit differences in proteins involved in 5-ALA metabolism, uptake, and transport.
- Large cohort studies using multi-omics approaches are warranted for a better understanding of molecular heterogeneity driving differential metabolism of 5-ALA.

**Importance of the Study**

Inter and intratumoral heterogeneity of HGGs makes their treatment difficult. Prognosis depends mainly on the extent of resection and 5-ALA fluorescence-guided surgery has proven its worth in this regard. However, there exists a subpopulation of tumors where 5-ALA is not effective. Genomics and metabolomics approaches have so far failed to offer a clear molecular picture driving this anomaly. In the current study, we have used the power of HR LC–MS based proteomics approaches to investigate whether the underlying ambiguity is a result of differential protein expression in various regions of the tumors. The study highlights the protein expression changes between differentially fluorescing regions from the same tumor. These findings indicate that large cohort-based studies using multi-OMICS approaches are warranted for a better understanding of 5-ALA metabolism at the molecular level.

High-grade gliomas (HGGs) comprise WHO grades 3 and 4 diffuse gliomas that affect the glial cells in the CNS. These are among the most aggressive forms of gliomas and recurrence is inevitable even in patients receiving treatment postsurgery. Patients usually survive on average only 15 months after its diagnosis and very few patients, ie, 3% can make it beyond 5 years. Major reasons for such low survival rates include difficulty to treat these tumors due to the tumor heterogeneity: both inter- and intra-tumoral,^1,2^ and high migratory ability making it impossible to surgically resect the tumors completely.^3,4^ The extent of resection (EOR) remains an important prognostic factor.^5^ Malignant gliomas are notorious for their diffuse nature and pose challenges in intraoperative delineation. Hence, the importance of adjuncts to increase the efficiency of surgery by enhancing the visualization of the tumor boundary has been on the rise in recent times. 5-ALA-induced tumor fluorescence is one such widely used technique in glioma surgery, and it relies on the metabolic conversion of 5-ALA into endogenous protoporphyrin IX (PpIX) which is a fluorophore.^6^

5-ALA is a metabolite belonging to the heme biosynthesis pathway. It is produced naturally after a condensation reaction between succinyl CoA (TCA cycle) and glycine in the presence of ALA synthase. After a series of enzymatic reactions, 5-ALA gives rise to PpIX, a photosensitive compound, which forms heme by the action of the enzyme ferrochelatase (FECH). In the case of glioma cells, the activity of this enzyme is affected leading to the accumulation of PpIX.^7^ During surgery, malignant tissues having accumulated PpIX, fluoresce red under UV light which differentiates them from healthy tissues. The excitation wavelengths are 405 nm and 633 nm. Other than dysfunction in FECH, high natural levels of 5-ALA, high expression of epidermal growth factor receptor (EGFR) and EGFRvIII, IDH1 gene mutational status, reduced NADPH levels, and glutaminase-2 expression are some of the other reasons for PpIX accumulation.^7–9^

Studies have shown that the degree of fluorescence correlates with the histological tumor type (strong with highly cellular and more malignant tumor phenotype). This property has been exploited clinically to identify regions of the highest tumor grade while sampling gliomas.^10,11^ Visualized fluorescence can sometimes be subjective and lead to variability in defining fluorescing regions with respect to (w.r.t) their biological grade. Objective assessment of fluorescence is possible using spectroscopic techniques and ex vivo PpIX assessment assays. The biometric study in our previous work revealed that even with objective assessment using PpIX assays, there exists a small but definite subpopulation of tumor cells with similar histological phenotypes but discordant metabolic/biochemical properties w.r.t accumulation of PpIX.^12^ In the current study, we extended the investigation further and have carried out proteomics analysis of high-grade glioma tissue samples resected using 5-ALA fluorescence-guided surgery to understand differences at the protein level driving differential fluorescence in these complex and heterogenous tumors.

## Materials and Methods

### Sample Collection

The study was approved by the Institutional Review Board (IRB) at Tata Memorial Hospital (TMC-IEC III—Project no.139) and Institute Ethics Committee (IEC) at IIT Bombay (IITB-IEC/2018/020). Written informed consent was obtained for all the participants in the study. The detailed protocol followed for collecting samples and biometric analysis to estimate the levels of PpIX has been mentioned in our previous work.^12^ Briefly, patients enrolled in the study were orally premedicated with 5-ALA (Gliolan, Medac GmBh, Germany; @ 20 mg/kg body) 4–5 h prior to surgery. Fluorescing tumor regions were observed using the BLUE 400 filter fitted to a OPMI Pentero microscope (Carl Zeiss, Oberkochen, Germany).

Surgeries were carried out by a team of surgeons with one lead surgeon (A.M.). Tumors were sampled based on regions of differing fluorescence quality within the viable tumor tissue (excluding necrosis). Some regions were also selected from the infiltrating zone. Depending on overall fluorescence distribution (homogenous or heterogenous), in some cases, multiple regions were sampled. The sampled differentially fluorescing regions were further divided into 3 parts where the first part was used for histopathology, the second part was used for biometric measurement of PpIX levels, and the last part was used for proteomics analysis.

Following objective assessment and estimation of PpIX levels, we categorized the samples as nonfluorescing (NF) with PpIX levels between 0 and 0.35 µg/g, weak fluorescing (WF) with PpIX levels between 0.36 and 0.79 µg/g and strongly fluorescing (SF) with PpIX levels between 0.80 and 5.81 µg/g. However, our proteomics study included samples only from the SF and NF regions and WF regions were not considered. All the regions of the tumors were evaluated for their histopathology by the lead pathologist (S.E). The fluorescence status was conveyed to the pathologist prior to the histopathological analysis, though the exact PpIX levels were unknown. Owing to the heterogeneity in the tumors, sub-regions of the same tumor were found to exhibit histological characteristics similar to grade III (nuclear atypia and high cellularity), grade IV (microvascular proliferation and necrosis in addition to nuclear atypia and high cellularity). The regions of these HGGs were classified as histologically grade III and histologically grade IV upon final evaluation. The proteomics evaluation of the samples was carried out after an objective assessment of PpIX levels and histopathology of the samples. A total of 12 fluorescent and 16 nonfluorescent regions from 11 patients was used for the proteomics study.

### Sample Preparation for Proteomics Analysis

All samples for proteomics experiments were processed at the Proteomics lab, IIT Bombay. Briefly, 30 mg of tissue was lysed by sonication at 40% amplitude for 2.5 min with 5 s pulse cycles in 300 µl of lysis buffer containing 8 M urea, 50 mM Tris pH 8.0, 75 mM NaCl, 1 mM MgCl_2_, 500 units of benzonase. Following this, the lysate was clarified by centrifugation at 8000 rpm, 4 °C for 15 min, and clear supernatant was split into multiple aliquots for storage at −80 °C until further use.

Protein lysates were quantified using the micro-bicinchoninic acid assay (micro-BCA assay). A total of 50 µg of protein lysate was subjected to in-solution digestion. The proteins were first reduced and then alkylated using 20 mM tris (2-carboxyethyl) phosphine (TCEP) and 37.5 mM iodoacetamide (IAA), respectively. The proteins were then subjected to digestion using trypsin (Pierce) at a ratio of 1:30 at 37 °C for 16 h. Following digestion, the peptides were desalted using in-house C18 zip-tips, dried, and stored for future use.

### Liquid Chromatography Coupled With Tandem Mass Spectrometry (LC–MS/MS)

The desalted and dried peptides were reconstituted in 0.1% FA for quantification using the scopes method.^13^ The peptides were separated through reverse phase chromatography using an Easy nLC 1200 system (Thermo Fisher Scientific). Briefly, 1 µg of the peptide was loaded onto the C18 nano viper trap column (Acclaim PepMap 100, 75 µm × 2 cm) and equilibrated at a flow rate of 5 µL/min. Peptides were then resolved on an analytical column (EASY-spray PepMap RSLC C18 reversed-phase column, 2 μm, 75 μm × 500 mm) at a flow rate of 300 nL/min over a 120 min gradient in solvent B (80% ACN in 0.1% FA).

The spectra were measured using the Orbitrap Fusion Tribrid MS platform (Thermo Fisher Scientific) operating in the positive ion mode. A full MS scan was performed at a resolution of 60 000 and the full scan ranged from 350 to 1700 *m*/*z*. The MS2 scan was carried out at a resolution of 15 000 using the TopN DDA method (*N* = 20). MS2 fragmentation was achieved through higher energy collisional dissociation (HCD) using a normalized collision energy (NCE) of 27. The mass window was set to 10 ppm with a dynamic exclusion duration of 40 s. Mass accuracy during the acquisition was ensured through the lock mass option using the polysiloxane species (*m*/*z* 445.12003). All the sample runs were randomized with each sample injected in triplicates. To account for any run-to-run variation resulting during the experiment, a common pool sample was injected after every 6 sample injections.

### Label-Free Quantification-Based Proteomics Data Analysis

Primary data analysis on the raw data files generated was performed with MaxQuant (v2.1.3.0) against the Uniprot human database proteome file (proteome ID: UP000005640) searched with the built-in Andromeda Search Engine of MaxQuant.^14^ Raw files were processed using Label Free Quantification (LFQ) parameters and setting label-type as “standard” with a multiplicity of 1 on the Orbitrap Fusion mode. Maximum missed cleavages were set to 2, carbamidomethylation of cysteine (+57.021464 Da) was set as a fixed modification, and oxidation of methionine (+15.994915 Da) was set as a variable modification. false discovery rate (FDR) was set to 1% for PSM, protein, and site decoy fraction to ensure high reliability of the protein detection. Decoy mode was set to “revert,” and the type of identified peptides was set to “unique + razor.” Further downstream analysis was carried out on the “proteins groups.txt” file obtained after MaxQuant analysis.

Proteins containing no unique peptides were filtered out from the dataset along with contaminants and reverse proteins. For each protein in the list, the median value of its abundance from the triplicates was considered. Statistical analysis of the samples was performed using ProTIGY (v1.0.1). The resulting dataset was log_2_ transformed and proteins with greater than 30% of missing values across all samples were filtered out. The dataset was then normalized using the Median-MAD option in ProTIGY. Based on IDH gene mutation status and fluorescence pattern, the high-grade glioma samples were divided into 4 subgroups—IDH wild type with fluorescence (IDH wt. Flu), IDH wild type without fluorescence (IDH wt. NonFlu), IDH mutant with fluorescence (IDH mt. Flu), and IDH mutant without fluorescence (IDH mt. NonFlu). To identify proteins with significant differences in the expression between the fluorescing and nonfluorescing regions, 2 sample *t*-tests were carried out for comparisons—(i) all IDH wt. Flu regions vs. all IDH wt. NonFlu regions and (ii) all IDH mt. Flu regions vs IDH mt. NonFlu regions.

### Gene Set Enrichment Analysis and Network visualization

To identify significantly enriched pathways in fluorescent and nonfluorescent regions of the tumors, we applied pre-ranked GSEA (GSEA 4.2.3) using FC values and p-value, with 1000 permutations for all the expressed proteins.^15^ The enrichment statistics were selected as weighted, with a meandiv normalization. Hallmark gene sets (H) and Curated Gene Set (C2: cgp and cp.reactome) from Human MSigDB collections were chosen as background databases. The biologically enriched pathways in the different subgroups of tumors were selected considering FDR *q* value < 0.05 and normalized enrichment score (NES) of ≥+1.5 or ≤−1.5 as cut-off. We then used ClueGO and CluePedia plug-in for Cytoscape software (Cytoscape 3.9.1) to build an interaction network containing genes that were core enriched in each biological pathway. Common entities from the list of significant proteins obtained from secondary data analysis were then highlighted in the network.

## Results

### Quality Control of Samples for Proteomic Analysis

All the samples used for the proteomics study were assessed for their quality before secondary analysis and subsequent statistical testing. The pooled sample that was injected every day was used to assess the performance of the instrument during the course of the study. Our analysis indicated a high level of consistency in the instrument performance which was evident from the reproducibility in the total number of proteins identified daily (Supplementary Figure 1A), low variability between pools (Supplementary Figure 1C), and high correlation between the pools (Supplementary Figure 1D).

After verifying consistency in instrument performance, we evaluated the quality of the individual patient samples. The evaluation was based on the total number of proteins identified in each individual mass spectrometric measurement and the consistency in the identified proteins among the triplicate measurements for every individual patient (Supplementary Figure 1B). Out of the 28 individual regions (12 fluorescent and 16 nonfluorescent) from 11 patients, only 17 regions (9 fluorescent and 8 nonfluorescent) from 8 patients passed our criteria for the study and were considered for further analysis. Supplementary Table 1 provides clinical information for the samples used in the final analysis and Supplementary Table 2 provides clinical information of the discarded samples along with reasons for their exclusion from the study.

### Histological Correlation With PpIX Heterogeneity

**Figure 1.**
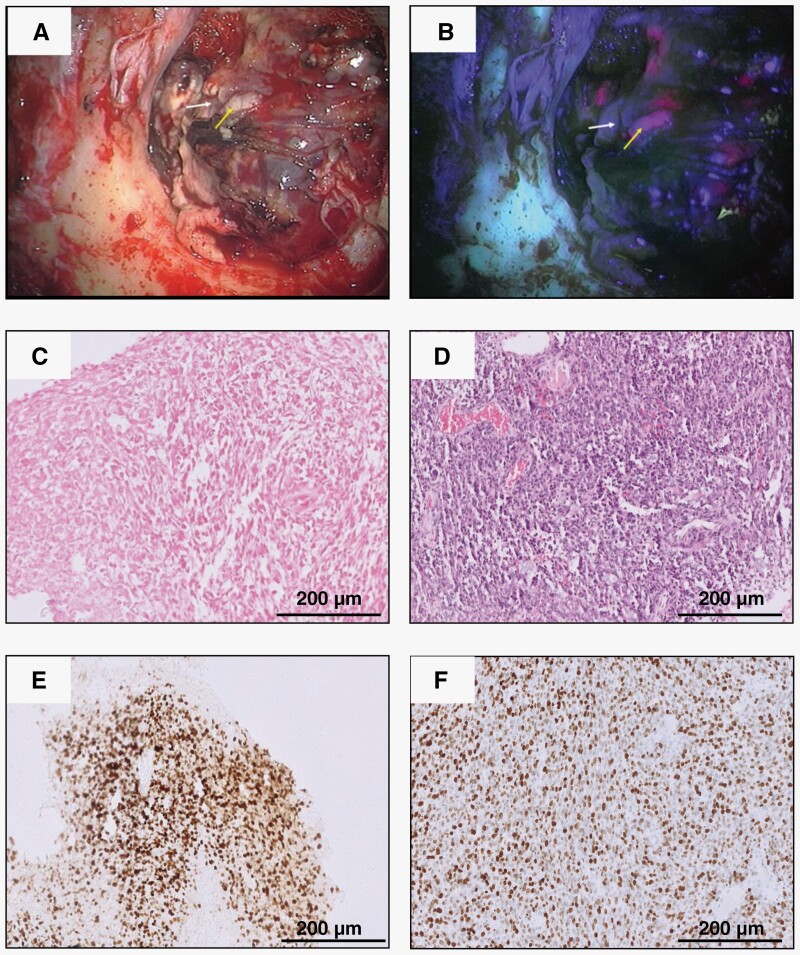
Intraoperative and histopathological images of tumors. (A) Intraoperative image of tumor as observed under the conventional white light setting, (B) Intraoperative image of tumor as observed under the blue light setting. The white arrow highlights the region of the tumor with weak fluorescence while yellow arrow indicates the region with strong fluorescence, (C) H&E staining of tumor from the fluorescing region of patient #3 as observed at 100× magnification, (D) H&E staining of tumor from the nonfluorescing region of patient #3 as observed at 100× magnification, (E) Ki-67 staining of the tumor from the fluorescing region of patient #3 as observed at 100× magnification, and (F) Ki-67 staining of the tumor from the nonfluorescing region of patient #3 as observed at 100× magnification.

The samples used in the study comprised of glioblastoma (IDH wt.) and grade 4 astrocytoma (IDH mt.) tumors. Samples with astrocytic morphology, with high-grade histological features (mitotic activity with microvascular proliferation and/necrosis), negative for IDH on IHC, and with retained ATRX protein expression were considered glioblastoma (IDH wt.). All the other samples were considered as high-grade astrocytoma (IDH mt.). For each case, individual biopsy samples were classified as histologically high-grade (non-necrotic regions with increased tumor cell density and mitotic activity) and histologically low grade (infiltrating low cell density regions). While we observed an expected concordant correlation between the histological grade and PpIX levels in most patients, 3 patients in the current study were observed to have PpIX levels discordant with the pathological grade. Nonfluorescing regions from patient nos. 1, 2, and 3 showed low levels of PpIX despite being histologically high grade. These samples were referred to as discordant samples in our study and investigated separately. Figure 1C–1F represents the histopathology of 1 representative patient exhibiting discordance in PpIX levels w.r.t tumor pathology.

### Proteomic Alterations in Fluorescing Versus Nonfluorescing Regions of Glioblastomas

**Figure 2.**
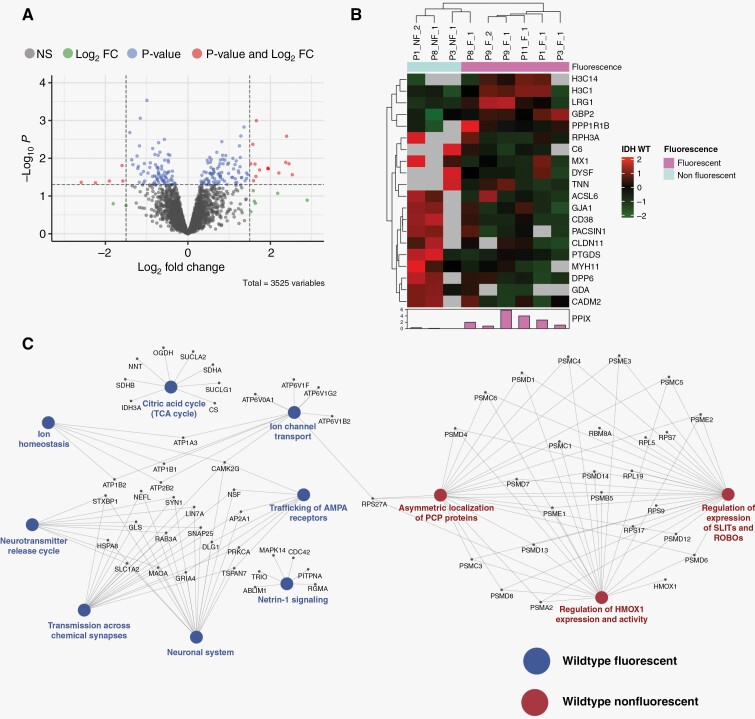
Differential proteomics analysis of differentially fluorescing regions of Glioblastomas. (A) Volcano plot showing differentially regulated proteins with proteins in red passing the *P*-value and log_2_ FC of ≤ 0.05 and ≥1.5, respectively. (B) Heatmap with significantly dysregulated proteins showing a distinct pattern between the 2 groups of samples. (C) Network indicating the complex interplay between differentially regulated pathways in the 2 groups of samples.

The label-free quantitative analysis of glioblastomas identified 20 differentially regulated proteins between the fluorescing and nonfluorescing regions. Of these, 5 proteins were down-regulated while 15 proteins were up-regulated in the nonfluorescing regions when compared to fluorescing regions (Supplementary Table 3). Proteins such as cell adhesion molecule 2 (CADM2), gap junction alpha-1 protein (Connexin-43), protein kinase C and casein kinase substrate in neurons protein 1 (Syndapin-1), Tenascin-N, Dysferlin-1 with well-known roles in cell adhesion and migration in Gliomas were found to be dysregulated in the comparison (Figure 2A). However, we could not find any significant differences between the expression of proteins related to the heme biosynthesis pathway.

GSEA analysis revealed pathways such as citric acid cycle, ion channel transport, Netrin-1 signaling, trafficking of AMPA receptors, and ion homeostasis to be enriched in the fluorescing regions of glioblastomas. Conversely, key pathways enriched in the nonfluorescing regions of these tumors included regulation of heme-oxygenase-1 expression and activity, localization of PCP proteins, and regulation and expression of SLITS and ROBOs (Figure 2C, Supplementary Figures 2 and 3). It may be argued that the statistical significance of the analysis could have been slightly skewed at the protein and pathway level due to the discordance seen in behavior of differentially fluorescing regions from patient no. 3 (Figure 2B).

### Proteomic Alterations in Differentially Fluorescing Regions of IDH Mutant High-Grade Gliomas

**Figure 3.**
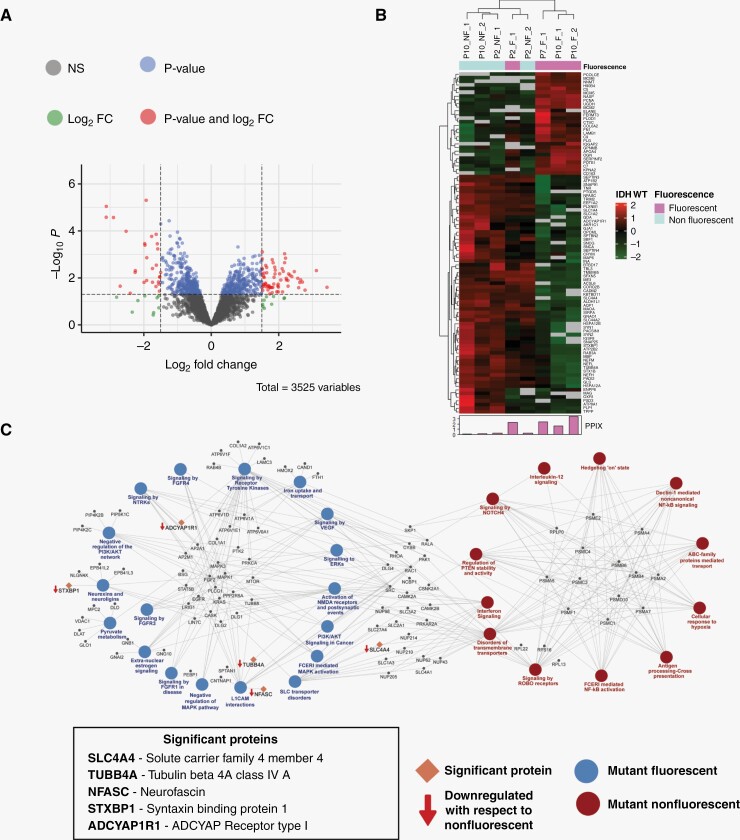
Differential proteomics analysis of differentially fluorescing regions of IDH mutant high-grade gliomas. (A) Volcano plot showing differentially regulated proteins with proteins in red passing the *P*-value and log_2_ FC of ≤.05 and ≥1.5, respectively. (B) Heatmap with significantly dysregulated proteins showing a distinct pattern between the 2 groups of samples. (C) Network indicating the complex interplay between differentially regulated pathways in the 2 groups of samples.

The label-free quantitative analysis of heterogeneously fluorescing IDH mutant high-grade gliomas identified 93 differentially regulated proteins (Supplementary Table 4). These proteins included proteins involved in transport of ions and amino acids (transporter proteins), heat shock proteins (HSPs), and cell adhesion, to name a few (Figure 3A and 3B).

GSEA analysis revealed dysregulation of interesting pathways not seen in the earlier comparison involving differentially fluorescing regions of glioblastomas. Key pathways enriched in the nonfluorescing regions included scavenging of heme by scavenger receptors, disorders in transmembrane receptors, signaling by NOTCH4, and cellular response to hypoxia. The fluorescing regions showed marked differences in pathways related to iron uptake and transport, signaling by fibroblast growth factor receptor, negative regulation of MAPK pathway, negative regulation of PI3-AKT pathway, and activation of NMDA receptors and postsynaptic events (Figure 3C, Supplementary Figures 4, 5, and 6).

### Comparative Analysis of Differentially Fluorescing Regions of Discordant Samples

**Figure 4.**
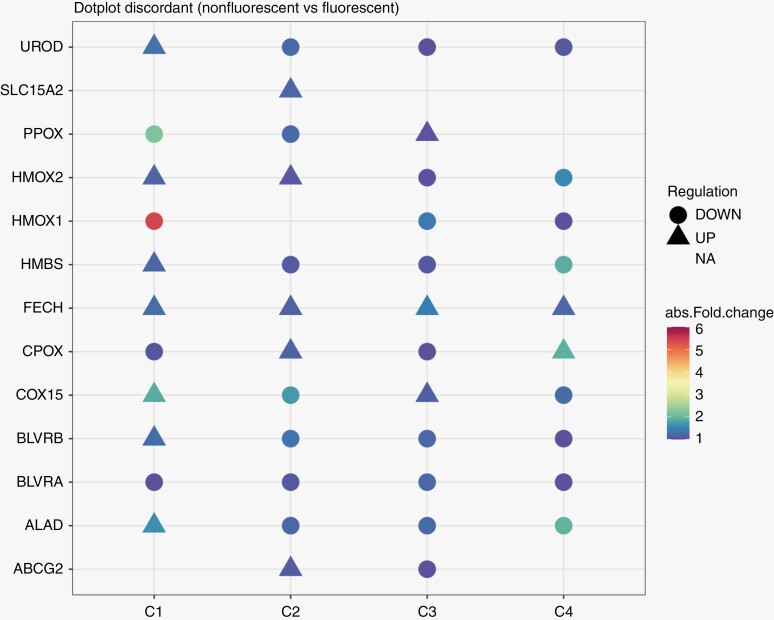
Dot plot for proteins involved in heme biosynthesis and 5-ALA uptake in transport. C1 indicates comparison of nonfluorescing region (NF1) with corresponding fluorescing region (F1) of patient no. 1, C2 indicates comparison with first nonfluorescing regions (NF2) with fluorescing region (F2) of patient no. 2, C3 indicates comparison of second nonfluorescing region (NF3) with fluorescing region (F2) of patient no. 2 and C4 indicates comparison of nonfluorescing region (NF4) with fluorescing region (F3) of patient no. 3.

As mentioned in Section 3.1, discordant samples in our study were investigated separately. Specifically, we set about investigating the expression of key proteins with roles in heme-biosynthesis and transport of 5-ALA in these samples to gain insights into the mechanisms driving differential fluorescence in these seemingly similar (w.r.t corresponding fluorescing regions) areas of tumors (Figure 4). Trends of expression for key proteins such as heme oxygenase-1 (HMOX1) and ferrochelatase (FECH) were observed to be similar though to varying degrees in different comparisons. However, 2 proteins–solute carrier family 15 member 2 (SLC15A2, also known as PEPT2) and ATP binding cassette subfamily G member 2 (ABCG2) with roles in transport of 5-ALA and PpIX,^16,17^ respectively in and out of the cell were found to be regulated differently in these patients. It was interesting to note that SLC15A2 expression was seen in only one nonfluorescing region from patient 2 but was absent in the other nonfluorescing region from the same patient. The expression of ABCG2 on the other hand was seen to be different in both nonfluorescing regions, ie, the protein was upregulated in nonfluorescing region 1 but downregulated in nonfluorescing region 2 w.r.t to the single fluorescing region from the same patient. In addition to this, the trends of protein expression for other accessory proteins were found to be different for patient no. 1 in comparison to patient nos. 2 and 3, suggesting a heterogeneity in the metabolism of 5-ALA upon entry into these tumors.

## Discussion

The use of 5-ALA in surgical procedures for glial tumors has significantly improved survival outcomes in patients over the past few years.^18–20^ Formed by the condensation of succinyl coA and glycine in a reaction catalyzed by the enzyme ALA synthase, this metabolite belongs naturally to the heme biosynthesis pathway. After a series of enzymatic reactions, 5-ALA gives rise to PpIX, a photosensitive compound, which forms heme by the action of the enzyme ferrochelatase (FECH). In the case of glioma cells, the activity of this enzyme is affected leading to the accumulation of PpIX.^7^ Other than dysfunction in FECH, high natural levels of 5-ALA, high expression of epidermal growth factor receptor (EGFR) and EGFRvIII, IDH1 gene mutation, reduced NADPH and glutaminase-2 expression are some of the factors influencing PpIX accumulation.^7–9^ Despite this, there exists a certain degree of uncertainty as far as the intensity of fluorescence is concerned, bringing a level of randomness to its use routinely as a surgical adjunct and for therapy.

Our previous work revealed the existence of a subpopulation with discordant biological properties. In this study, we found that surgical specimens varying in their fluorescence profile resected following 5-ALA fluorescence-guided surgery in high-grade gliomas, expressed distinct proteomic expression profiles. The expression profile of nonfluorescent tumor tissue from both IDH wt. and mt. subgroups was enriched for proteins associated with cellular functions and biological pathways characteristic of the neural subgroup of glioblastoma. This finding was in accordance with the study previously reported by Bonnin et al. using a genomics approach.^21^ In a manner consistent with the high degree of heterogeneity observed in high-grade gliomas, our results demonstrate that sections within glioma tumors that respond differently to 5-ALA, are proteomically distinct.

Many other factors with potential roles in influencing 5-ALA metabolism and PpIX levels in the tumors have been extensively reported. These include special transporter proteins like PEPT1 and PEPT2,^22–24^ ABCG2 and ATP Binding Cassette Subfamily B Member 6 (ABCB6) protein transporter.^25^ High activity of ABCG2 can potentially result in lower intensity of fluorescence following 5-ALA administration. The role of the ABCB6 protein transporter in the regulation of PpIX metabolism in leukemic cells has been demonstrated. In surgically resected glioma samples, the levels of mRNA of ABCB6 were found to be higher than the levels in normal brain tissue. However, there are conflicting reports regarding the role of ABCB6 in PpIX accumulation and this area requires further exploration.

The role of Fe^2+^ ions in the regulation of heme-oxygenase-1 (HMOX1) and ferrochelatase has been extensively reported.^26^ The high activity of HMOX1 results in a large production of Fe^2+^ ions. Importantly, the levels of these ions regulate the efficiency of the enzyme FECH.^27,28^ The rapid depletion of heme itself may alter the enzymatic activity in favor of increased PpIX metabolism by FECH. The net result of this is the increase in the rate of heme synthesis due to Fe^2+^ and PpIX, thus causing a reduction in fluorescence intensity in the tumor cells. We observed heme scavenging pathway to be enriched in the nonfluorescing regions of IDH mt. subgroup while the pathway for iron uptake and transport pathway was enriched in the fluorescing regions of the IDH mt. subgroup. Additionally, we observed a concordance in the expression levels of HMOX1 and FECH levels in the discordant patient samples. This heterogeneity highlights the potential limitation of 5-ALA as a diagnostic tool which can lead to missing high-grade tumor areas. More importantly, understanding the underlying biological mechanism of this heterogeneity can allow us to potentially modulate the same to improve outcomes.

Our study using LC–MS/MS has for the first time indicated the presence of heterogeneity at the proteome level driving differential metabolism of 5-ALA in individual patients. However, the role of factors such as MGMT promoter methylation, p53 status, and ATRX status in these tumors could not be studied due to the low sample size of the study. Future studies using a substantially higher number of samples and using other omics approaches in combination with proteomics can offer crucial insights into the interplay of proteins, genes, and metabolites at multiple levels driving heterogeneity in 5-ALA metabolism. In addition to this, since high-grade gliomas are very heterogeneous, often containing different glioma molecular subtypes (neural, proneural, mesenchymal, or classical) within the same tumor, an approach using multiple fluorophores and optical biomarkers may be more effective in detecting these highly heterogeneous tumors intraoperatively. Combining 2 or more intraoperative detection methods, such as ALA, fluorescein, anti- EGFR, or anti-VEGFR targeted fluorophores, quantum dot nanoparticles, or other agents, holds potential in targeting different subtypes of high-grade gliomas simultaneously, thereby improving intraoperative tumor detection and gross total resection. In this regard, an improved biological understanding will help guide technological developments to advance the field of fluorescence-guided neurosurgical oncology in the future.

## Supplementary Material

vdad065_suppl_Supplementary_Figures

vdad065_suppl_Supplementary_Tables

## Data Availability

The mass spectrometry proteomics data have been deposited to the ProteomeXchange Consortium via the PRIDE partner repository with the dataset identifier PXD039170.

## References

[1] Syafruddin SE , Nazarie WFWM, Moidu NA, Soon BH, Mohtar MA. Integration of RNA-Seq and proteomics data identifies glioblastoma multiforme surfaceome signature. BMC Cancer. 2021;21(1):850. 34301218 10.1186/s12885-021-08591-0 PMC8306276

[2] Georgescu MM , Islam MZ, Li Y, Traylor J, Nanda A. Novel targetable FGFR2 and FGFR3 alterations in glioblastoma associate with aggressive phenotype and distinct gene expression programs. Acta Neuropathol Commun. 2021;9(1):69. 33853673 10.1186/s40478-021-01170-1 PMC8048363

[3] Aldape K , Brindle KM, Chesler L, et al. Challenges to curing primary brain tumours. Nat Rev Clin Oncol. 2019;16(8):509–520. 30733593 10.1038/s41571-019-0177-5 PMC6650350

[4] Mazurek M , Grochowski C, Litak J, et al. Recent trends of microRNA significance in pediatric population glioblastoma and current knowledge of micro RNA function in glioblastoma multiforme. Int J Mol Sci . 2020;21(9):E3046. 10.3390/ijms21093046 PMC7246719 32349263

[5] Brown TJ , Brennan MC, Li M, et al. Association of the extent of resection with survival in glioblastoma: a systematic review and meta-analysis. JAMA Oncol. 2016;2(11):1460–1469. 27310651 10.1001/jamaoncol.2016.1373 PMC6438173

[6] Stummer W , Pichlmeier U, Meinel T, et al; ALA-Glioma Study Group. Fluorescence-guided surgery with 5-aminolevulinic acid for resection of malignant glioma: a randomised controlled multicentre phase III trial. Lancet Oncol. 2006;7(5):392–401. 16648043 10.1016/S1470-2045(06)70665-9

[7] Kim S , Kim JE, Kim YH, et al. Glutaminase 2 expression is associated with regional heterogeneity of 5-aminolevulinic acid fluorescence in glioblastoma. Sci Rep. 2017;7(1):12221. 28939850 10.1038/s41598-017-12557-3 PMC5610329

[8] Saito K , Hirai T, Takeshima H, et al. Genetic factors affecting intraoperative 5-aminolevulinic acid-induced fluorescence of diffuse gliomas. Radiol Oncol. 2017;51(2):142–150. 28740449 10.1515/raon-2017-0019 PMC5514654

[9] Fontana AO , Piffaretti D, Marchi F, et al. Epithelial growth factor receptor expression influences 5-ALA induced glioblastoma fluorescence. J Neurooncol. 2017;133(3):497–507. 28500562 10.1007/s11060-017-2474-0 PMC5537329

[10] Widhalm G , Kiesel B, Woehrer A, et al. 5-Aminolevulinic acid induced fluorescence is a powerful intraoperative marker for precise histopathological grading of gliomas with non-significant contrast-enhancement. PLoS One. 2013;8(10):e76988e76988. 24204718 10.1371/journal.pone.0076988 PMC3800004

[11] Widhalm G , Wolfsberger S, Minchev G, et al. 5-Aminolevulinic acid is a promising marker for detection of anaplastic foci in diffusely infiltrating gliomas with nonsignificant contrast enhancement. Cancer. 2010;116(6):1545–1552. 20108311 10.1002/cncr.24903

[12] Moiyadi A , Shetty P, Sridhar E, et al. Objective assessment of intraoperative tumor fluorescence reveals biological heterogeneity within glioblastomas: a biometric study. J Neurooncol. 2020;146(3):477–488. 32020478 10.1007/s11060-019-03338-1

[13] Scopes RK. Measurement of protein by spectrophotometry at 205 nm. Anal Biochem. 1974;59(1):277–282. 4407487 10.1016/0003-2697(74)90034-7

[14] Tyanova S , Temu T, Cox J. The MaxQuant computational platform for mass spectrometry-based shotgun proteomics. Nat Protoc. 2016;11(12):2301–2319. 27809316 10.1038/nprot.2016.136

[15] Subramanian A , Tamayo P, Mootha VK, et al. Gene set enrichment analysis: a knowledge-based approach for interpreting genome-wide expression profiles. Proc Natl Acad Sci U S A. 2005;102(43):15545–15550. 16199517 10.1073/pnas.0506580102 PMC1239896

[16] Teng L , Nakada M, Zhao SG, et al. Silencing of ferrochelatase enhances 5-aminolevulinic acid-based fluorescence and photodynamic therapy efficacy. Br J Cancer. 2011;104(5):798–807. 21304523 10.1038/bjc.2011.12 PMC3048207

[17] Müller P , Abdel Gaber SA, Zimmermann W, Wittig R, Stepp H. ABCG2 influence on the efficiency of photodynamic therapy in glioblastoma cells. J Photochem Photobiol B. 2020;210:111963. 32795847 10.1016/j.jphotobiol.2020.111963

[18] Gandhi S , Tayebi Meybodi A, Belykh E, et al. Survival outcomes among patients with high-grade glioma treated with 5-aminolevulinic acid-guided surgery: a systematic review and meta-analysis. Front Oncol. 2019;9:620. 31380272 10.3389/fonc.2019.00620 PMC6652805

[19] Díez Valle R , Tejada Solis S, Idoate Gastearena MA, et al. Surgery guided by 5-aminolevulinic fluorescence in glioblastoma: volumetric analysis of extent of resection in single-center experience. J Neurooncol. 2011;102(1):105–113. 20607351 10.1007/s11060-010-0296-4

[20] Della Puppa A , Ciccarino P, Lombardi G, et al. 5-Aminolevulinic acid fluorescence in high grade glioma surgery: surgical outcome, intraoperative findings, and fluorescence patterns. Biomed Res Int. 2014;2014:232561. 24804203 10.1155/2014/232561 PMC3997860

[21] Almiron Bonnin DA , Havrda MC, Lee MC, et al. Characterizing the heterogeneity in 5-aminolevulinic acid-induced fluorescence in glioblastoma. J Neurosurg. 2019;132(6):1706–1714. 31125970 10.3171/2019.2.JNS183128

[22] Hagiya Y , Endo Y, Yonemura Y, et al. Pivotal roles of peptide transporter PEPT1 and ATP-binding cassette (ABC) transporter ABCG2 in 5-aminolevulinic acid (ALA)-based photocytotoxicity of gastric cancer cells in vitro. Photodiagnosis Photodyn Ther. 2012;9(3):204–214. 22959800 10.1016/j.pdpdt.2011.12.004

[23] Ishizuka M , Abe F, Sano Y, et al. Novel development of 5-aminolevurinic acid (ALA) in cancer diagnoses and therapy. Int Immunopharmacol. 2011;11(3):358–365. 21144919 10.1016/j.intimp.2010.11.029

[24] Piffaretti D , Burgio F, Thelen M, et al. Protoporphyrin IX tracer fluorescence modulation for improved brain tumor cell lines visualization. J Photochem Photobiol B. 2019;201:111640. 31734545 10.1016/j.jphotobiol.2019.111640

[25] Zhao SG , Chen XF, Wang LG, et al. Increased expression of ABCB6 enhances protoporphyrin IX accumulation and photodynamic effect in human glioma. Ann Surg Oncol. 2013;20(13):4379–4388. 22688660 10.1245/s10434-011-2201-6

[26] Wang W , Tabu K, Hagiya Y, et al. Enhancement of 5-aminolevulinic acid-based fluorescence detection of side population-defined glioma stem cells by iron chelation. Sci Rep. 2017;7:42070. 28169355 10.1038/srep42070 PMC5294410

[27] Wilks A , Heinzl G. Heme oxygenation and the widening paradigm of heme degradation. Arch Biochem Biophys. 2014;544:87–95. 24161941 10.1016/j.abb.2013.10.013 PMC6476305

[28] Nimura T , Weinstein PR, Massa SM, Panter S, Sharp FR. Heme oxygenase-1 (HO-1) protein induction in rat brain following focal ischemia. Brain Res Mol Brain Res. 1996;37(1-2):201–208. 8738152 10.1016/0169-328x(95)00315-j

