# Supplementary material for: Deep proteome investigation of high-grade gliomas reveals heterogeneity driving differential metabolism of 5-aminolevulinic acid
Source: Neurooncol Adv. 2023 Jun 16;5(1):vdad065. doi: 10.1093/noajnl/vdad065 (PMC10290514; doi:10.1093/noajnl/vdad065)
Supplement: vdad065_suppl_Supplementary_Figures [file vdad065_suppl_supplementary_figures.docx]

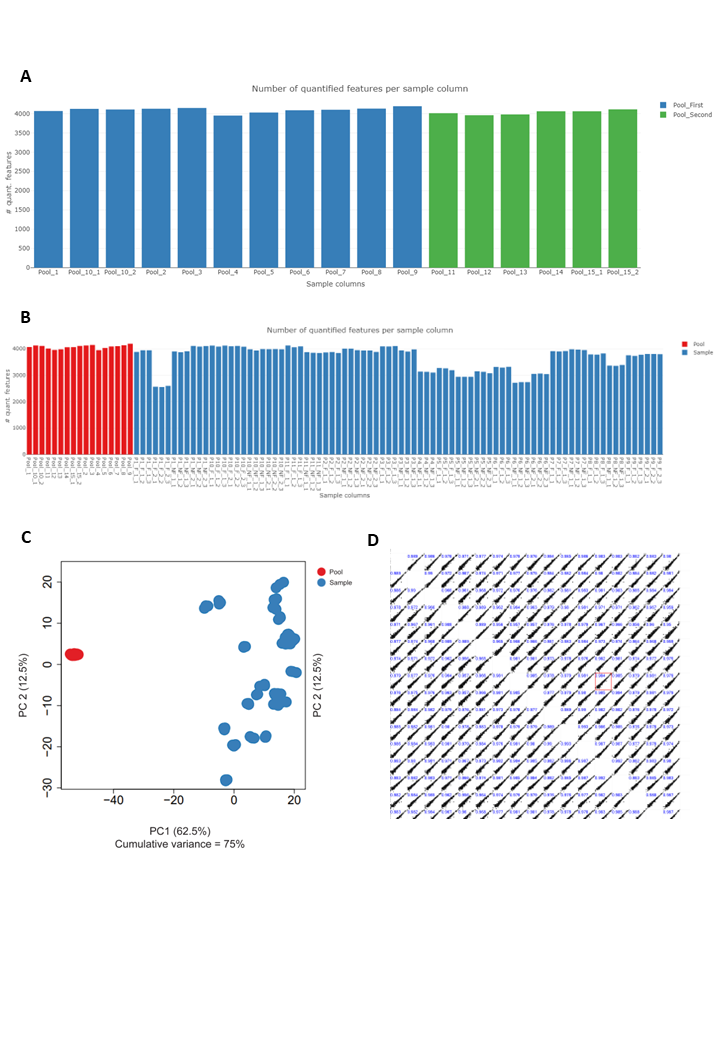


**Supplementary Figure 1:** Quality control of samples used in the proteomics study. A) Consistency in protein numbers from pool sample injected in two batches. B) Consistency and high reproducibility in protein numbers across all injections when the samples were run in triplicates. C) Clustering of all the sample injections using Principal component analysis (PCA) indicates a high reproducibility in instrument performance as evidenced from a single cluster for pool sample, and high degree of heterogeneity among the individual samples noticeable from their ubiquitous spread in the plot. D) Correlation plots for all the pool sample injections shows high correlation indicating a high degree of reproducibility in instrument performance throughout the experiment.


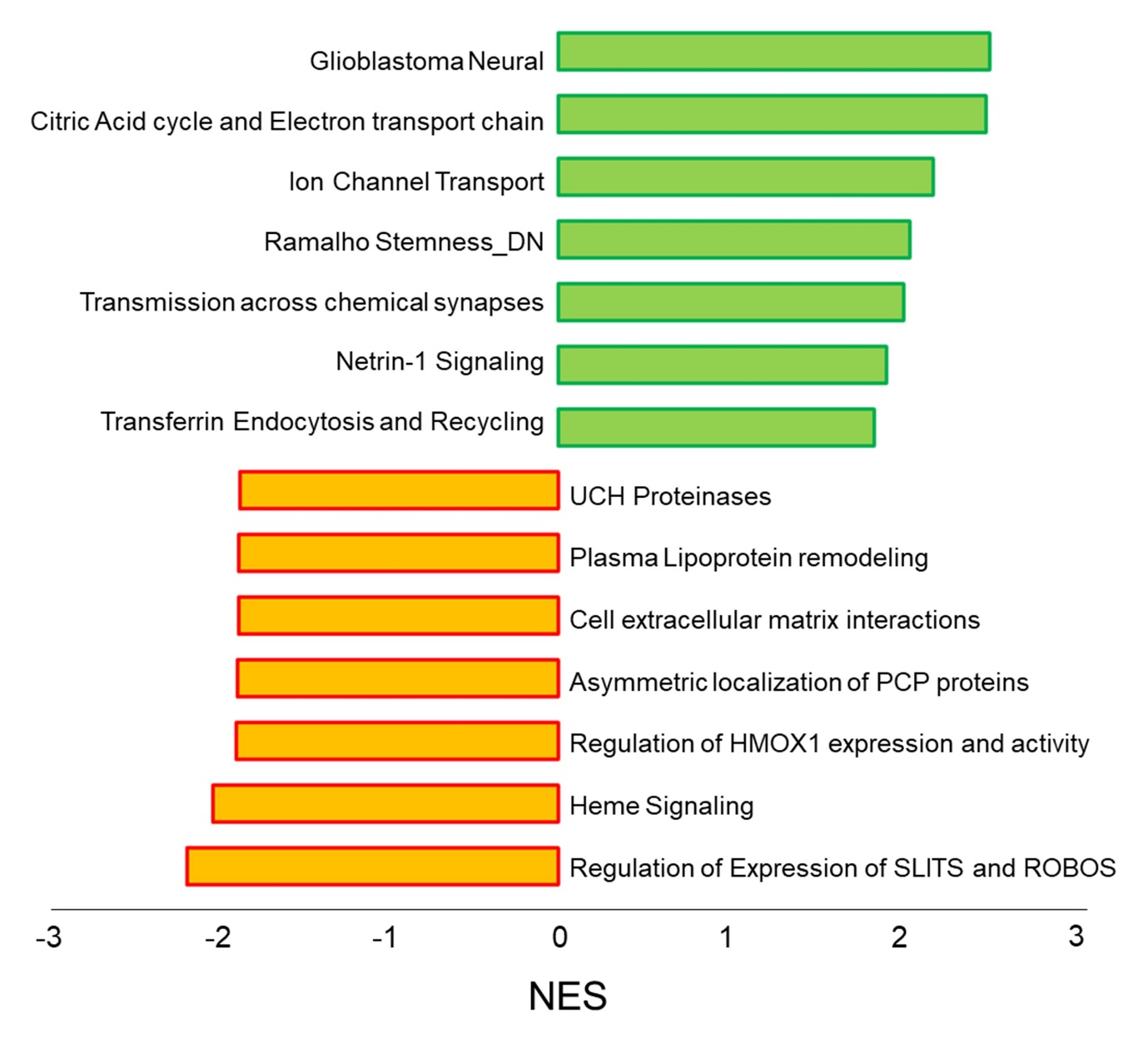


**Supplementary Figure 2:** Horizontal bar chart indicating Normalised Enrichment Scores (NES) for key pathways (FDR q value<0.05 and NES of ≥+1.5 or ≤-1.5) identified from GSEA analysis of IDH wt. samples. The bars in green indicate pathways that are positively enriched while the bars in yellow indicate pathways negatively enriched following comparison between the IDH wt. Flu and NonFlu regions.


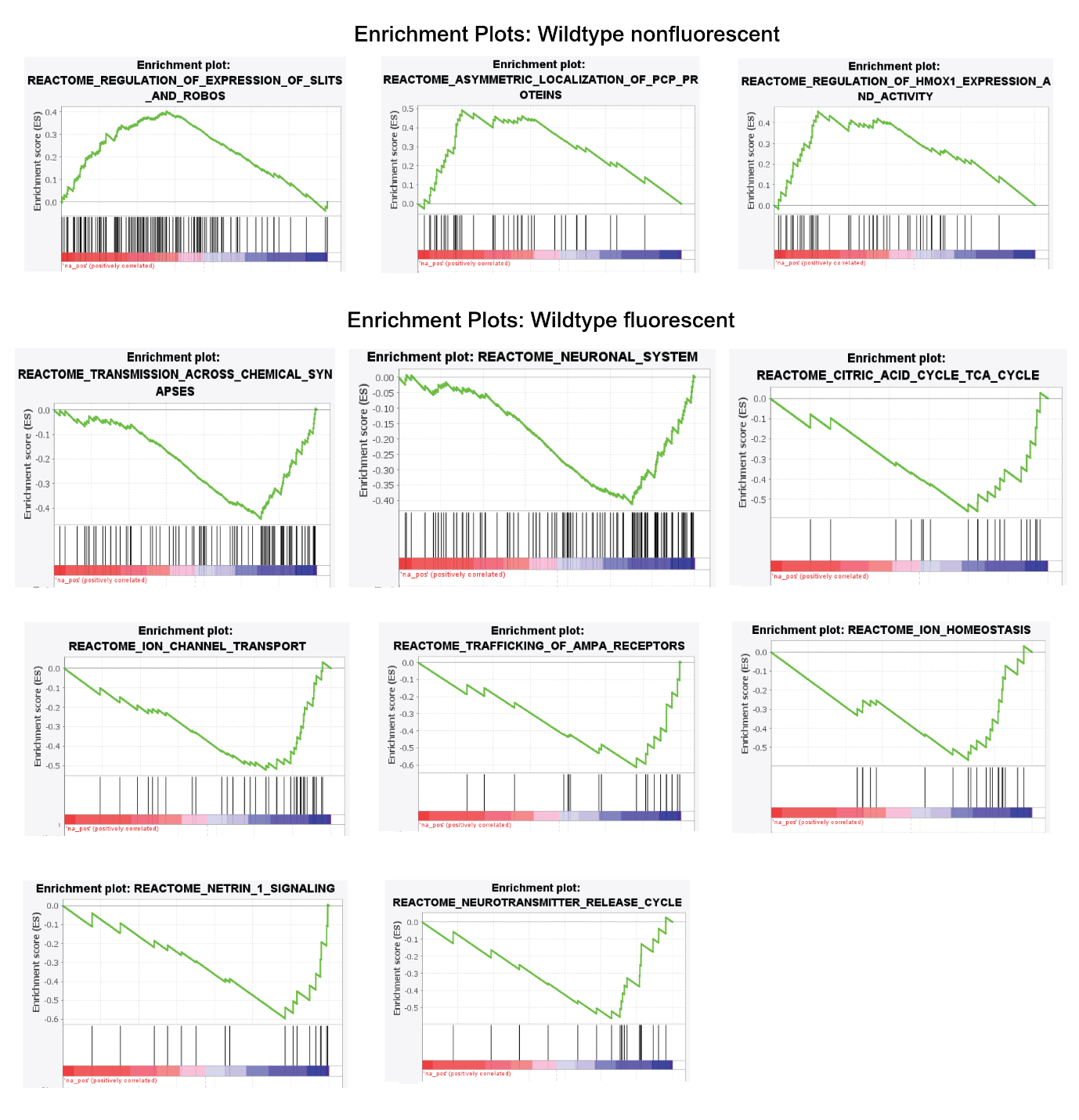


**Supplementary Figure 3:** Random walk plots for enriched pathways from GSEA analysis of IDH wt. Flu and NonFlu regions.


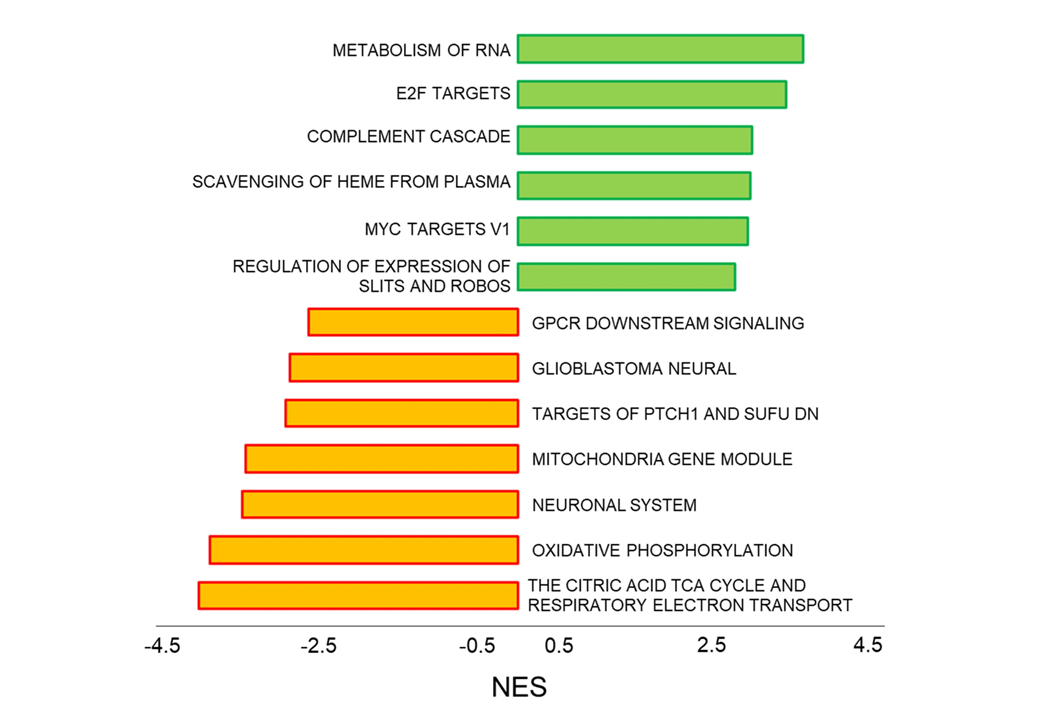


**Supplementary Figure 4:** Horizontal bar chart indicating Normalised Enrichment Scores (NES) for key pathways (FDR q value<0.05 and NES of ≥+1.5 or ≤-1.5) identified from GSEA analysis of IDH mt. samples. The bars in green indicate pathways that are positively enriched while the bars in yellow indicate pathways negatively enriched following comparison between the IDH mt. Flu and NonFlu regions.


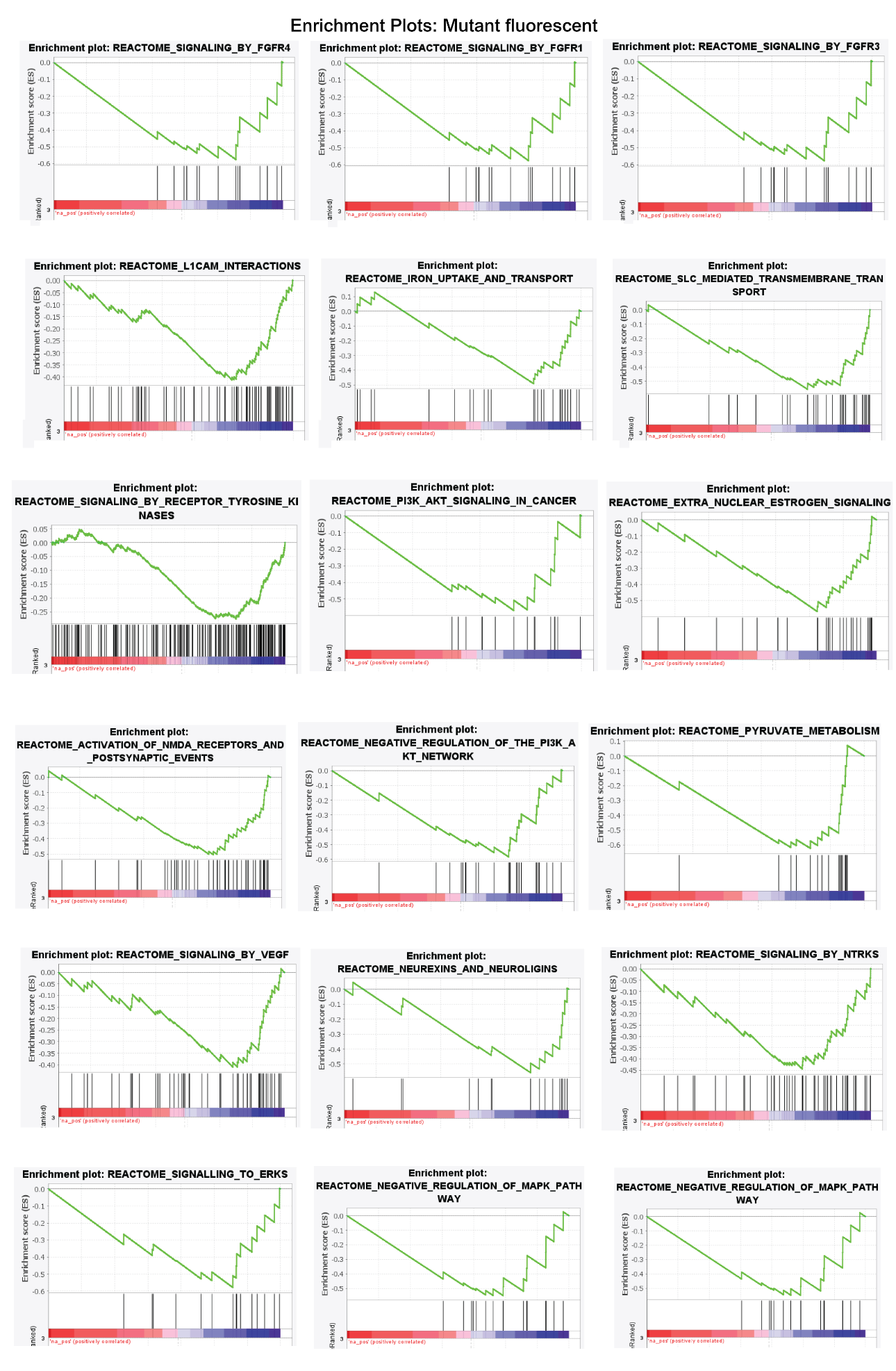
**Supplementary Figure 5:** Random walk plots for enriched pathways from GSEA analysis of IDH mt. Flu regions.


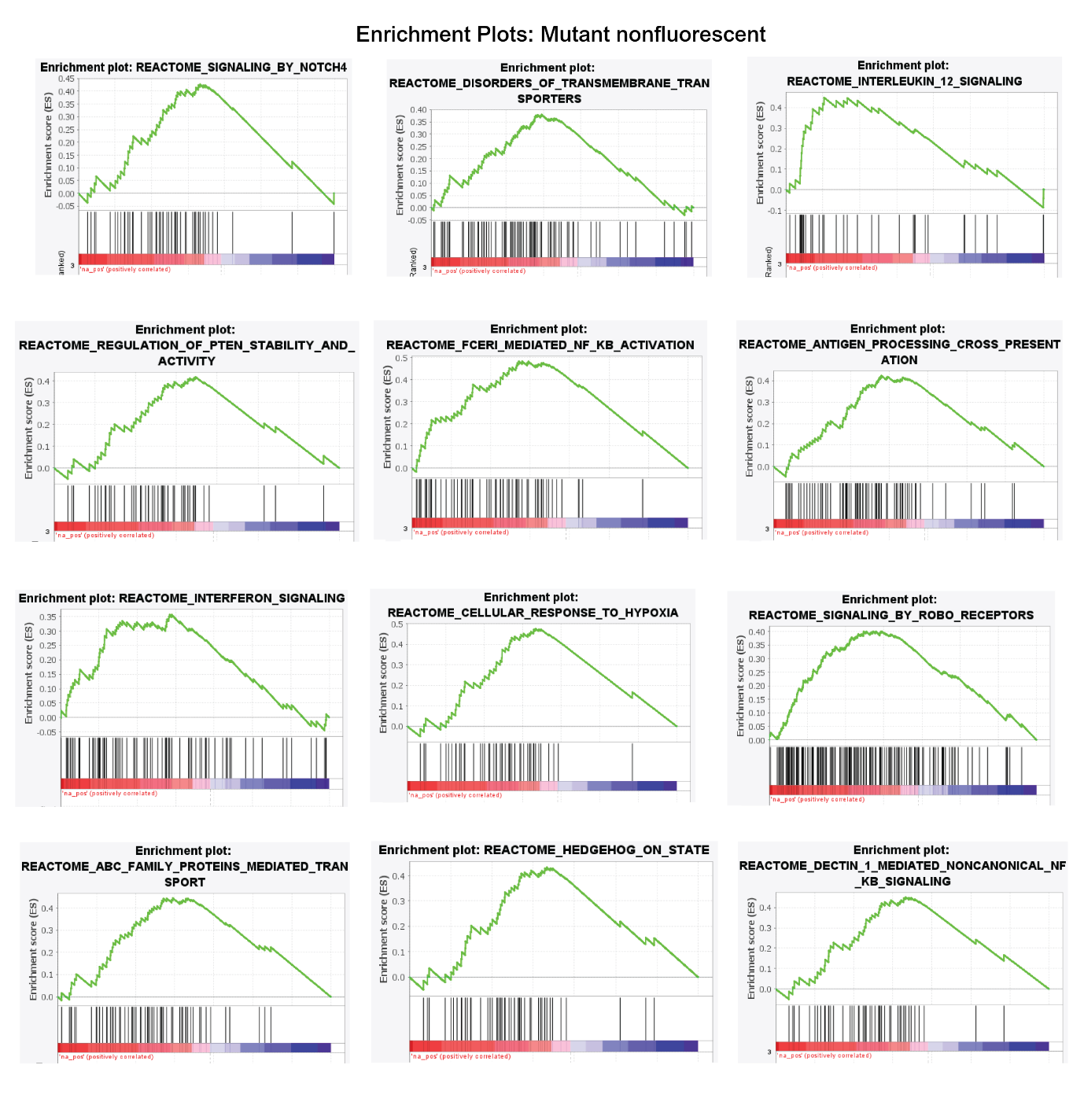


**Supplementary Figure 6:** Random walk plots for enriched pathways from GSEA analysis of IDH mt. NonFlu regions.
