# Supplementary material for: Deep proteome investigation of high-grade gliomas reveals heterogeneity driving differential metabolism of 5-aminolevulinic acid
Source: Neurooncol Adv. 2023 Jun 16;5(1):vdad065. doi: 10.1093/noajnl/vdad065 (PMC10290514; doi:10.1093/noajnl/vdad065)
Supplement: vdad065_suppl_Supplementary_Tables [file vdad065_suppl_supplementary_tables.docx]

**Supplementary table 1:** Clinical information for samples used in the proteomics study.

| **Patient#** | **Sex** | **Age** | **Site** | **IDH1 R132H** | **p53** | **ATRX** | **MGMT** | **Tumor Grade** | **Classification** | **Sample Annotation** | **Grade Subregion** | **Fluorescence status** | **PpIX levels** | **MIB Labeling index** |
| --- | --- | --- | --- | --- | --- | --- | --- | --- | --- | --- | --- | --- | --- | --- |
| Patient# 1 | Male | 59 | L-Frontal | IDH wt. | Negative | Not Retained | Unmethylated | WHO Grade 4 | Glioblastoma | P1_F_1 | Grade 4 | Fluorescent | 2.71 | 15-20% |
|  |  |  |  |  |  |  |  |  |  | P1_NF_2 | Grade 3 | Non-Fluorescent | 0.28 | 10-12% |
| Patient# 2 | Male | 38 | L-Posterior Frontal | IDH mt. | Negative | Not Retained | Methylated | WHO Grade 4 | IDH mutant Astrocytoma, Grade 4 | P2_F_1 | Grade 2-3 | Fluorescent | 2.31 | 4-6% |
|  |  |  |  |  |  |  |  |  |  | P2_NF_1 | Grade 3 | Non-Fluorescent | 0.2 | 5-7% |
|  |  |  |  |  |  |  |  |  |  | P2_NF_2 | Grade 3 | Non-Fluorescent | 0.26 | 8-10% |
| Patient# 3 | Male | 30 | R-Inferior Frontal | IDH wt. | Negative | Retained | Methylated | WHO Grade 4 | Glioblastoma | P3_F_1 | Grade 4 | Fluorescent | 1.11 | 50-60% |
|  |  |  |  |  |  |  |  |  |  | P3_NF_1 | Grade 4 | Non-Fluorescent | 0 | 50-60% |
| Patient# 7 | Female | 34 | R-Posterior Frontal | IDH mt. | Positive | Not Retained | Unmethylated | WHO Grade 4 | IDH mutant Astrocytoma, Grade 4 | P7_F_1 | Grade 4 | Fluorescent | 2.4 | 8-10% |
| Patient# 8 | Female | 66 | R-Temporo Parietal | IDH wt. | Negative | Retained | Not determined | WHO Grade 4 | Glioblastoma | P8_F_1 | Grade 4 | Fluorescent | 1.99 | 6-8% |
|  |  |  |  |  |  |  |  |  |  | P8_NF_1 | Grade 2-3 | Non-Fluorescent | 0.1 | Non contributory |
| Patient# 9 | Male | 59 | L-Temporal | IDH wt. | Positive | Retained | Unmethylated | WHO Grade 4 | Glioblastoma | P9_F_1 | Grade 4 | Fluorescent | 0.8 | 15-20% |
|  |  |  |  |  |  |  |  |  |  | P9_F_2 | Grade 4 | Fluorescent | 5.81 | 20-25% |
| Patient# 10 | Female | 24 | R-Parietal | IDH mt. | Positive | Not Retained | Not determined | WHO Grade 4 | IDH mutant Astrocytoma, Grade 4 | P10_F_1 | Grade 4 | Fluorescent | 1.67 | 60-70% |
|  |  |  |  |  |  |  |  |  |  | P10_F_2 | Grade 4 | Fluorescent | 3.46 | 60-70% |
|  |  |  |  |  |  |  |  |  |  | P10_NF_1 | Grade 3 | Non-Fluorescent | 0.08 | 15-20% |
|  |  |  |  |  |  |  |  |  |  | P10_NF_2 | Grade 3 | Non-Fluorescent | 0.1 | 8-10% |
| Patient# 11 | Female | 66 | R- Parieto Occipital | IDH wt. | Positive | Retained | Unmethylated | WHO Grade 4 | Glioblastoma | P11_F_1 | Grade 4 | Fluorescent | 4 | 15-20% |

**Supplementary table 2:** List of samples and reasons for their exclusion from the proteomics study.

| **Patient #** | **Sex** | **Age** | **Site** | **IDH1 R132H** | **p53** | **ATRX** | **MGMT** | **Tumor Grade** | **Classification** | **Sample Annotation** | **Grade Subregion** | **Fluorescence status** | **PpIX levels** | **MIB Labeling index** | **Reason for exclusion** |
| --- | --- | --- | --- | --- | --- | --- | --- | --- | --- | --- | --- | --- | --- | --- | --- |
| Patient# 1 | Male | 59 | L- Frontal | IDH wt. | Negative | Not Retained | Unmethylated | WHO Grade IV | Glioblastoma | P1_F_2 | Grade 4 | Fluorescent | 4.6 | 15-20% | Poor sample quality |
|  |  |  |  |  |  |  |  |  |  | P1_NF_1 | Grade 4 | Non-Fluorescent | 0.31 | Non contributory | High Necrosis |
| Patient# 4 | Female | 45 | L- Inferior Frontal | IDH mt. | Postive | Retained | NA | Grade III | IDH mutant Astrocytoma Grade 3 | P4_NF_1 | Grade 3 | Non-Fluorescent | 0.22 | 3-5% | Poor sample quality |
| Patient# 5 | Male | 35 | Bifrontal Corpus callosal | IDH mt. | Positive | Not Retained | NA | WHO Grade IV | IDH mutant Astrocytoma Grade 4 | P5_NF_1 | Grade 4 | Non-Fluorescent | 0.17 | 8-10% | Poor sample quality |
|  |  |  |  |  |  |  |  |  |  | P5_NF_2 | Grade 4 | Non-Fluorescent | 0.13 | 8-10% | Poor sample quality |
|  |  |  |  |  |  |  |  |  |  | P5_F_1 | Grade 4 | Fluorescent | 2.1 | 15-20% | Poor sample quality |
| Patient# 6 | Female | 59 | L- Temporo Parietal | IDH wt. | Positive | Retained | Unmethylated | WHO Grade IV | Glioblastoma | P6_F_1 | Grade 4 | Fluorescent | 2.3 | Non contributory | Non-Neoplastic Reactive |
|  |  |  |  |  |  |  |  |  |  | P6_NF_1 | Grade 4 | Non-Fluorescent | 0.16 | 4-6% | Poor sample quality |
|  |  |  |  |  |  |  |  |  |  | P6_NF_2 | Grade 4 | Non-Fluorescent | 0.11 | Non contributory | High Necrosis |
| Patient# 7 | Female | 34 | R- Posterior Frontal | IDH mt. | Positive | Not retained | Unmethylated | WHO Grade IV | IDH mutant Astrocytoma, Grade 4 | P7_NF_1 | Grade 4 | Non-Fluorescent | 0.13 | 6-8% | High Necrosis |
| Patient# 11 | Female | 66 | R- Parieto Occipital | IDH wt. | Positive | Retained | Unmethylated | WHO Grade IV | Glioblastoma | P11_NF_1 | NA | Non-Fluorescent | 0.17 | Non contributory | Non-Neoplastic Reactive |

**Supplementary table 3:** List of statistically significant dysregulated proteins in IDH wt. Flu and IDH wt. NonFlu regions of HGGs.

| **Uniprot ID** | **Protein Name** | **Avg.Abundance (Flu)** | **Avg.Abundance (NonFlu)** | **Log FC (NonFlu/Flu)** | **p-value** | **Std. Dev** | **Std. error** | **Present in all samples** |
| --- | --- | --- | --- | --- | --- | --- | --- | --- |
| Q71DI3 | Histone H3.1 | 25.83 | 23.25 | -2.59 | 4.337E-02 | 1.510 | 0.616 | No |
| P68431 | Dipeptidyl aminopeptidase-like protein 6 | 25.98 | 23.74 | -2.24 | 4.460E-02 | 1.875 | 0.625 | Yes |
| Q9UD71 | Long-chain-fatty-acid--CoA ligase 6 | 25.22 | 23.31 | -1.91 | 4.005E-02 | 1.382 | 0.489 | No |
| P32456 | Guanylate-binding protein 2 | 24.10 | 22.50 | -1.60 | 1.557E-02 | 1.153 | 0.384 | No |
| P02750 | Histone H3.2 | 23.99 | 22.42 | -1.58 | 4.003E-02 | 1.292 | 0.431 | No |
| Q9Y2J0 | Rabphilin-3A | 22.04 | 23.57 | 1.52 | 1.398E-02 | 0.741 | 0.280 | No |
| P42658 | Interferon-induced GTP-binding protein Mx1 | 22.80 | 24.37 | 1.57 | 4.277E-03 | 0.901 | 0.319 | No |
| Q9Y2T3 | Myosin-11 | 22.10 | 23.69 | 1.59 | 3.924E-02 | 1.223 | 0.499 | No |
| O75923 | ADP-ribosyl cyclase/cyclic ADP-ribose hydrolase 1 | 23.38 | 25.01 | 1.63 | 1.427E-02 | 0.795 | 0.301 | No |
| Q9UKU0 | Claudin-11 | 22.14 | 23.79 | 1.65 | 3.022E-02 | 1.144 | 0.404 | No |
| P28907 | Complement component C6 | 23.32 | 24.98 | 1.66 | 1.011E-03 | 0.881 | 0.312 | No |
| P41222 | Tenascin-N | 22.58 | 24.32 | 1.74 | 2.040E-02 | 1.297 | 0.432 | No |
| P20591 | Cell adhesion molecule 2 | 22.35 | 24.29 | 1.94 | 1.896E-02 | 1.275 | 0.451 | No |
| Q9UQP3 | Guanine deaminase | 23.37 | 25.32 | 1.94 | 1.822E-02 | 0.982 | 0.371 | No |
| P35749 | Protein kinase C and casein kinase substrate in neurons protein 1 | 21.52 | 23.47 | 1.95 | 1.894E-02 | 1.436 | 0.508 | No |
| Q8N3J6 | Leucine-rich alpha-2-glycoprotein | 22.93 | 25.13 | 2.20 | 2.455E-02 | 1.497 | 0.529 | No |
| Q9BY11 | Protein phosphatase 1 regulatory subunit 1B | 22.13 | 24.50 | 2.37 | 1.281E-02 | 1.514 | 0.572 | No |
| P13671 | Dysferlin | 22.95 | 25.34 | 2.39 | 2.607E-03 | 1.097 | 0.448 | No |
| P17302 | Prostaglandin-H2 D-isomerase | 23.16 | 25.61 | 2.45 | 1.397E-02 | 1.564 | 0.553 | No |
| O75508 | Gap junction alpha-1 protein | 23.52 | 26.05 | 2.53 | 2.727E-02 | 1.755 | 0.663 | No |

**Supplementary table 4:** List of statistically significant dysregulated proteins in IDH mt. Flu and IDH mt. NonFlu regions of HGGs.

| **Uniprot ID** | **Protein Name** | **Avg.Abundance (Flu)** | **Avg.Abundance (NonFlu)** | **Log FC (NonFlu/Flu)** | **p-value** | **Std. Dev** | **Std. error** | **Present in all samples** |
| --- | --- | --- | --- | --- | --- | --- | --- | --- |
| O43301 | Heat shock 70 kDa protein 12A | 23.62 | 25.12 | 1.50 | 2.561E-02 | 1.182 | 0.418 | Yes |
| O60641 | Clathrin coat assembly protein AP180 | 23.49 | 25.34 | 1.85 | 1.823E-02 | 1.407 | 0.497 | Yes |
| O60701 | UDP-glucose 6-dehydrogenase | 24.61 | 23.04 | -1.57 | 1.488E-02 | 1.160 | 0.410 | Yes |
| O75891 | Cytosolic 10-formyltetrahydrofolate dehydrogenase | 22.75 | 24.30 | 1.55 | 8.715E-03 | 1.091 | 0.386 | Yes |
| P00747 | Plasminogen | 24.80 | 23.21 | -1.59 | 5.851E-04 | 0.957 | 0.339 | Yes |
| P02751 | Fibronectin | 25.51 | 23.76 | -1.74 | 1.529E-02 | 1.296 | 0.458 | Yes |
| P07942 | Laminin subunit beta-1 | 24.83 | 23.30 | -1.53 | 9.193E-03 | 1.082 | 0.383 | Yes |
| P08697 | Alpha-2-antiplasmin | 24.08 | 22.10 | -1.97 | 3.468E-04 | 1.177 | 0.416 | Yes |
| P12036 | Neurofilament heavy polypeptide | 22.62 | 25.27 | 2.65 | 1.208E-02 | 1.941 | 0.686 | Yes |
| P12110 | Collagen alpha-2 | 25.37 | 23.83 | -1.54 | 3.666E-02 | 1.275 | 0.451 | Yes |
| P14415 | Sodium/potassium-transporting ATPase subunit beta-2 | 23.76 | 25.51 | 1.75 | 2.601E-02 | 1.383 | 0.489 | Yes |
| P21397 | Amine oxidase [flavin-containing] A | 23.05 | 24.73 | 1.68 | 2.233E-02 | 1.300 | 0.460 | Yes |
| P37840 | Alpha-synuclein | 23.35 | 24.85 | 1.50 | 6.715E-03 | 1.033 | 0.365 | Yes |
| P49321 | Nuclear autoantigenic sperm protein | 24.56 | 23.01 | -1.56 | 1.842E-02 | 1.178 | 0.417 | Yes |
| P61764 | Syntaxin-binding protein 1 | 24.29 | 25.91 | 1.62 | 2.943E-03 | 1.052 | 0.372 | Yes |
| P78324 | Tyrosine-protein phosphatase non-receptor type substrate 1 | 22.86 | 25.04 | 2.18 | 2.248E-03 | 1.405 | 0.497 | Yes |
| Q16352 | Alpha-internexin | 23.50 | 25.78 | 2.28 | 2.246E-02 | 1.777 | 0.628 | Yes |
| Q8TD22 | Sideroflexin-5 | 22.59 | 24.86 | 2.27 | 1.282E-02 | 1.669 | 0.590 | Yes |
| Q9C040 | Tripartite motif-containing protein 2 | 22.96 | 24.53 | 1.57 | 6.294E-03 | 1.074 | 0.380 | Yes |
| Q9UQ03 | Coronin-2B | 22.93 | 24.74 | 1.81 | 1.681E-03 | 1.145 | 0.405 | Yes |
| A6NE02 | BTB/POZ domain-containing protein 17 | 22.61 | 24.33 | 1.72 | 2.378E-02 | 1.283 | 0.485 | No |
| O15020 | Spectrin beta chain, non-erythrocytic 2 | 23.21 | 25.05 | 1.83 | 2.276E-03 | 1.181 | 0.418 | No |
| O43157 | Plexin-B1 | 22.60 | 24.46 | 1.86 | 9.411E-03 | 1.323 | 0.468 | No |
| O43236 | Septin-4 | 22.16 | 24.04 | 1.88 | 3.484E-03 | 1.243 | 0.439 | No |
| O76070 | Gamma-synuclein | 22.04 | 24.03 | 1.99 | 2.462E-02 | 1.496 | 0.565 | No |
| O94811 | Tubulin polymerization-promoting protein | 23.99 | 25.50 | 1.52 | 4.269E-03 | 1.008 | 0.356 | No |
| O94819 | Kelch repeat and BTB domain-containing protein 11 | 22.06 | 24.76 | 2.70 | 1.511E-02 | 1.935 | 0.732 | No |
| O94856 | Neurofascin | 22.90 | 25.12 | 2.22 | 1.032E-02 | 1.599 | 0.565 | No |
| O94925 | Glutaminase kidney isoform, mitochondrial | 22.85 | 24.83 | 1.98 | 1.855E-02 | 1.510 | 0.534 | No |
| O95248 | Myotubularin-related protein 5 | 22.51 | 24.02 | 1.51 | 7.850E-04 | 0.921 | 0.325 | No |
| P01031 | Complement C5 | 22.99 | 21.35 | -1.64 | 2.330E-02 | 1.219 | 0.461 | No |
| P02686 | Myelin basic protein | 22.94 | 26.37 | 3.43 | 2.511E-02 | 2.725 | 0.963 | No |
| P02748 | Complement component C9 | 24.06 | 22.09 | -1.97 | 4.032E-04 | 1.145 | 0.433 | No |
| P04350 | Tubulin beta-4A chain | 25.03 | 27.06 | 2.03 | 6.709E-03 | 1.406 | 0.497 | No |
| P06727 | Apolipoprotein A-IV | 24.90 | 22.37 | -2.53 | 1.036E-04 | 1.467 | 0.519 | No |
| P07196 | Neurofilament light polypeptide | 23.55 | 26.33 | 2.78 | 3.322E-02 | 2.288 | 0.809 | No |
| P07197 | Neurofilament medium polypeptide | 24.17 | 26.76 | 2.59 | 2.613E-02 | 2.064 | 0.730 | No |
| P08246 | Neutrophil elastase | 24.91 | 22.21 | -2.70 | 2.248E-02 | 1.849 | 0.924 | No |
| P09471 | Guanine nucleotide-binding protein G | 25.01 | 26.65 | 1.64 | 3.593E-03 | 1.084 | 0.383 | No |
| P0CG38 | POTE ankyrin domain family member I | 25.72 | 24.12 | -1.60 | 3.524E-04 | 0.949 | 0.336 | No |
| P10643 | Complement component C7 | 23.39 | 21.75 | -1.63 | 1.400E-04 | 0.945 | 0.334 | No |
| P12004 | Proliferating cell nuclear antigen | 24.83 | 22.57 | -2.26 | 5.844E-03 | 1.551 | 0.548 | No |
| P17302 | Gap junction alpha-1 protein | 23.59 | 25.28 | 1.68 | 3.256E-02 | 1.372 | 0.485 | No |
| P17600 | Synapsin-1 | 23.66 | 25.56 | 1.90 | 4.690E-03 | 1.230 | 0.465 | No |
| P20336 | Ras-related protein Rab-3A | 23.30 | 24.87 | 1.57 | 9.582E-03 | 1.111 | 0.393 | No |
| P20774 | Mimecan | 24.98 | 23.06 | -1.92 | 1.289E-02 | 1.343 | 0.508 | No |
| P20916 | Myelin-associated glycoprotein | 21.15 | 24.27 | 3.12 | 4.881E-03 | 1.930 | 0.863 | No |
| P29972 | Aquaporin-1 | 24.06 | 25.61 | 1.55 | 2.310E-02 | 1.203 | 0.425 | No |
| P33992 | DNA replication licensing factor MCM5 | 27.00 | 25.25 | -1.74 | 3.985E-03 | 1.115 | 0.422 | No |
| P40261 | Nicotinamide N-methyltransferase | 24.55 | 21.44 | -3.11 | 9.011E-06 | 1.711 | 0.765 | No |
| P41222 | Prostaglandin-H2 D-isomerase | 23.10 | 25.09 | 1.99 | 3.925E-02 | 1.451 | 0.592 | No |
| P41586 | Pituitary adenylate cyclase-activating polypeptide type I receptor | 22.97 | 24.86 | 1.89 | 6.754E-03 | 1.256 | 0.475 | No |
| P43004 | Excitatory amino acid transporter 2 | 23.54 | 26.00 | 2.46 | 5.393E-03 | 1.684 | 0.595 | No |
| P43007 | Neutral amino acid transporter A | 23.25 | 25.31 | 2.06 | 1.237E-02 | 1.442 | 0.545 | No |
| P49736 | DNA replication licensing factor MCM2 | 24.96 | 23.03 | -1.93 | 4.972E-06 | 1.045 | 0.395 | No |
| P52292 | Importin subunit alpha-1 | 24.53 | 21.63 | -2.90 | 2.680E-05 | 1.615 | 0.610 | No |
| P53634 | Dipeptidyl peptidase 1 | 24.00 | 22.37 | -1.63 | 2.879E-03 | 1.020 | 0.386 | No |
| P60201 | Myelin proteolipid protein | 25.67 | 27.19 | 1.52 | 3.378E-02 | 1.248 | 0.441 | No |
| P60880 | Synaptosomal-associated protein 25 | 23.94 | 25.55 | 1.62 | 1.846E-02 | 1.170 | 0.442 | No |
| P61266 | Syntaxin-1B | 22.94 | 25.27 | 2.33 | 7.639E-03 | 1.636 | 0.579 | No |
| Q01814 | Plasma membrane calcium-transporting ATPase 2 | 23.18 | 25.38 | 2.20 | 1.445E-03 | 1.392 | 0.492 | No |
| Q02809 | Procollagen-lysine,2-oxoglutarate 5-dioxygenase 1 | 23.76 | 21.50 | -2.25 | 4.892E-03 | 1.446 | 0.590 | No |
| Q04828 | Aldo-keto reductase family 1 member C1 | 22.10 | 24.07 | 1.97 | 2.201E-02 | 1.462 | 0.553 | No |
| Q05639 | Elongation factor 1-alpha 2 | 23.89 | 25.44 | 1.55 | 1.896E-02 | 1.176 | 0.416 | No |
| Q12788 | Transducin beta-like protein 3 | 23.42 | 24.92 | 1.50 | 3.870E-03 | 0.991 | 0.350 | No |
| Q13576 | Ras GTPase-activating-like protein IQGAP2 | 23.93 | 22.43 | -1.50 | 5.846E-03 | 0.911 | 0.407 | No |
| Q14566 | DNA replication licensing factor MCM6 | 24.51 | 21.40 | -3.12 | 2.624E-05 | 1.724 | 0.771 | No |
| Q14894 | Ketimine reductase mu-crystallin | 22.94 | 24.55 | 1.61 | 1.681E-02 | 1.207 | 0.427 | No |
| Q14956 | Transmembrane glycoprotein NMB | 25.52 | 23.53 | -1.99 | 1.550E-02 | 1.391 | 0.568 | No |
| Q14982 | Opioid-binding protein/cell adhesion molecule | 22.89 | 24.53 | 1.64 | 6.050E-03 | 1.125 | 0.398 | No |
| Q15113 | Procollagen C-endopeptidase enhancer 1 | 22.08 | 20.56 | -1.53 | 8.987E-03 | 0.946 | 0.386 | No |
| Q16798 | NADP-dependent malic enzyme, mitochondrial | 22.84 | 24.79 | 1.94 | 3.896E-02 | 1.633 | 0.577 | No |
| Q58FF6 | Putative heat shock protein HSP 90-beta 4 | 24.53 | 22.12 | -2.40 | 4.508E-02 | 1.801 | 0.735 | No |
| Q6PI78 | Transmembrane protein 65 | 22.73 | 24.45 | 1.71 | 2.131E-02 | 1.321 | 0.467 | No |
| Q6UWR7 | Glycerophosphocholine cholinephosphodiesterase ENPP6 | 21.92 | 24.54 | 2.62 | 1.558E-02 | 1.884 | 0.712 | No |
| Q86UX7 | Fermitin family homolog 3 | 23.63 | 21.72 | -1.91 | 1.386E-03 | 1.203 | 0.425 | No |
| Q86VB7 | Scavenger receptor cysteine-rich type 1 protein M130 | 23.94 | 22.18 | -1.77 | 1.709E-04 | 1.003 | 0.379 | No |
| Q8IWA5 | Choline transporter-like protein 2 | 22.73 | 24.90 | 2.18 | 9.384E-04 | 1.348 | 0.477 | No |
| Q8N3J6 | Cell adhesion molecule 2 | 23.12 | 25.69 | 2.56 | 1.039E-02 | 1.773 | 0.670 | No |
| Q8N573 | Oxidation resistance protein 1 | 21.74 | 23.53 | 1.79 | 2.556E-02 | 1.351 | 0.511 | No |
| Q92752 | Tenascin-R | 23.83 | 25.94 | 2.11 | 3.943E-02 | 1.780 | 0.629 | No |
| Q92777 | Synapsin-2 | 23.11 | 25.24 | 2.14 | 5.307E-03 | 1.400 | 0.529 | No |
| Q969P0 | Immunoglobulin superfamily member 8 | 23.03 | 24.77 | 1.75 | 2.511E-02 | 1.313 | 0.496 | No |
| Q96JE9 | Microtubule-associated protein 6 | 22.36 | 24.46 | 2.10 | 4.049E-03 | 1.353 | 0.511 | No |
| Q96MM6 | Heat shock 70 kDa protein 12B | 23.02 | 24.53 | 1.51 | 1.538E-03 | 0.913 | 0.345 | No |
| Q9BY11 | Protein kinase C and casein kinase substrate in neurons protein 1 | 22.45 | 24.74 | 2.29 | 6.767E-03 | 1.531 | 0.579 | No |
| Q9NYI0 | PH and SEC7 domain-containing protein 3 | 21.48 | 23.21 | 1.73 | 4.800E-02 | 1.427 | 0.539 | No |
| Q9UH03 | Neuronal-specific septin-3 | 23.80 | 25.44 | 1.64 | 2.390E-02 | 1.279 | 0.452 | No |
| Q9UKU0 | Long-chain-fatty-acid--CoA ligase 6 | 22.42 | 24.22 | 1.81 | 4.262E-02 | 1.460 | 0.552 | No |
| Q9Y2J8 | Protein-arginine deiminase type-2 | 23.10 | 24.92 | 1.82 | 2.551E-02 | 1.441 | 0.510 | No |
| Q9Y2Q0 | Phospholipid-transporting ATPase IA | 21.52 | 23.08 | 1.56 | 1.955E-02 | 1.191 | 0.421 | No |
| Q9Y2T3 | Guanine deaminase | 22.09 | 24.48 | 2.39 | 8.782E-03 | 1.628 | 0.615 | No |
| Q9Y6R1 | Electrogenic sodium bicarbonate cotransporter 1 | 23.42 | 25.65 | 2.23 | 1.229E-02 | 1.560 | 0.589 | No |
